# An update on the impact of SARS-CoV-2 pandemic public awareness on cancer patients' COVID-19 vaccine compliance: Outcomes and recommendations

**DOI:** 10.3389/fpubh.2022.923815

**Published:** 2022-07-22

**Authors:** Lina Souan, Maher A. Sughayer, Maha Abu Alhowr, Khawlah Ammar, Sara Al Bader

**Affiliations:** Department of Pathology and Laboratory Medicine, King Hussein Cancer Center, Amman, Jordan

**Keywords:** survey, COVID-19, cancer patients, vaccine, SARS-CoV-2

## Abstract

**Background:**

Aside from the pandemic's negative health effects, the world was confronted with public confusion since proper communication and favorable decisions became an ongoing challenge. As a result, the public's perceptions were influenced by what they knew, the many sources of COVID-19 information, and how they interpreted it. With cancer patients continuing to oppose COVID-19 vaccines, we sought to investigate the COVID-19 pandemic and vaccine sources of this information in adult cancer patients, which either helped or prevented them from taking the vaccine. We also assessed the relevance and impact of their oncologists' recommendations in encouraging them to take the vaccine.

**Methods:**

From June to October 2021, an online survey was conducted at King Hussein Cancer Center. A total of 441 adult cancer patients took part in the study. Patients who had granted their consent were requested to complete an online questionnaire, which was collected using the SurveyMonkey questionnaire online platform. Descriptive analysis was done for all variables. The association between categorical and continuous variables was assessed using the Pearson Chi-square and Fisher Exact.

**Results:**

Our results showed that 75% of the patients registered for the COVID-19 vaccine, while 12% refused vaccination. The majority of participants acquired their information from news and television shows, whereas (138/441) got their information through World Health Organization websites. Because the SARS-CoV-2 vaccines were made in such a short period, 54.7 % assumed the vaccines were unsafe. Only 49% of the patients said their oncologists had informed them about the benefits of SARS-CoV-2 vaccines.

**Conclusions:**

We found that SARS-CoV-2 vaccine hesitancy in cancer patients might be related to misinformation obtained from social media despite the availability of supportive scientific information on the vaccine's benefits from the physicians. To combat misleading and unreliable social media news, we recommend that physicians use telehealth technology to reach out to their patients in addition to their face-to-face consultation, which delivers comprehensive, clear, and high-quality digital services that guide and help patients to better understand the advantages of COVID-19 vaccines.

## Introduction

Coronavirus Disease 2019 (COVID-19) began in Wuhan, China, in 2019 and was caused by a novel strain of coronaviruses called severe acute respiratory syndrome coronavirus 2 (SARS-CoV-2). In March 2020, the World Health Organization (WHO) announced COVID-19 as a worldwide pandemic, with cases ranging from asymptomatic to symptomatic infections with mild, moderate, or severe symptoms (1, 2). By the middle of October 2021, more than 239 million cases of SARS-CoV-2 infections were confirmed worldwide, and nearly 4.87 million deaths had been declared (3).

Since the outbreak of the COVID-19 pandemic began, national and international efforts have been taken to develop effective vaccines against SARS-CoV-2, and the development of vaccines has become the most realistic chance for the world to prevent the transmission of the virus and hence, return to normality (4).

Jordan reported 856,450 cases and 10,986 deaths until October 31^st^, 2021, accounting for about 5.2% of all confirmed cases and 3.6% of all deaths in the WHO Eastern Mediterranean Region (EMR) (5). Jordan was also one of the first 40 nations to get the vaccines, thus the Jordanian Ministry of Health began a vaccination campaign on January 13^th^, 2021, targeting healthcare workers, individuals with chronic illnesses, and those over the age of 60 (6). According to a survey conducted in Jordan between December 2^nd^ and December 29^th^, 2020 (before the start of the vaccine campaign), 72.3 % of Jordanians were willing to receive COVID-19 immunization, and COVID-19 risk of infection was significantly associated with vaccine acceptance (7). Immunocompromised patients, particularly cancer patients, were given special attention because, when infected with the SARS-CoV-2 virus, they have a higher risk of needing mechanical ventilation and admission to the intensive care unit (ICU), and the mortality rates are higher than people without cancer (8–10). COVID-19-related mortality rates among cancer patients are as high as 25.6 % (11). Consequently, national and international efforts emerged to develop practical guidelines to assist healthcare institutions in decreasing cancer patients' exposure to SARS-CoV-2. The most common guideline, derived from Cancer Care Ontario, involved prioritizing clinical management of cancer patients during the pandemic and thus reducing the impact of the pandemic upon healthcare workers and hospitals (12, 13).

Aside from the pandemic's negative health effects, the world faced another issue which was the spread of COVID-19 misinformation, hence increasing the likelihood of worse outcomes for vulnerable groups, such as cancer patients. As a result, public confusion emerged globally since proper communication and favorable decisions became an ongoing challenge (14).

COVID-19 infection had indeed a negative impact on the psychology of cancer patients who had higher levels of stress and anxiety due to their critical health situation (15, 16). For example, coronaphobia, which is described as an extreme fear of COVID-19 (17), was linked to a lot of vaccination skepticism. The lack of evidence on the safety and efficacy of the COVID-19 vaccination in cancer patients who were excluded from the early clinical trials created a knowledge gap, allowing misconceptions and false assumptions to emerge (18). In other words, the world faced a digital pandemic because of the tremendous amount of misinformation in different forms that have been spread worldwide. As a result, the attitudes and behaviors of the population depended on what they know, the various sources of COVID-19 information, and how they understand this information (19, 20).

Internationally, different studies have been conducted in countries such as England, Portugal, and Serbia to investigate the impact of COVID-19 and acceptance of COVID-19 vaccination in adult patients with cancer (21–23). Tunisia and Lebanon, for example, undertook similar investigations throughout the Arab region (24, 25). Both studies concluded that better communication with patients, whether directly through their oncologists or national campaigns and media, can lead to increased vaccination acceptability. Most patients appear to follow their oncologist's vaccine recommendations, indicating that the oncologist's influence is significant. A cross-sectional survey of 364 adult patients with cancer in Bosnia and Herzegovina found that 85.60% of study participants were willing to follow their oncologist's guidance on COVID-19 vaccination (26).

This issue dramatically increased the need to survey people's attitudes toward the COVID-19 pandemic to enhance the role of healthcare institutions and workers (HCWs) in raising awareness. This study aims to describe the attitudes, and knowledge, related to the COVID-19 pandemic and vaccination in adult patients with cancer, as obtained through survey analysis, to guide the best approaches for relaying COVID-19 information in this vulnerable population. We hypothesize that, despite physicians' advice to their patients to get the COVID-19 vaccine, cancer patients still refuse to get vaccinated because they depend on other sources of information other than their oncologists.

## Materials and methods

### Survey setting and design

This is an online survey analysis study conducted at King Hussein Cancer Center (KHCC) between June and October 2021 with 441 cancer patients treated at KHCC, regardless of their vaccination status or vaccine type taken. The SurveyMonkey questionnaire tool (San Mateo, California, USA) online platform was used to collect the data. All participants provided their informed consent, which was recorded electronically (https://www.surveymonkey.com/summary/eIPeQ x3KkZN1nVl4F6NhevYb2Osxfh_2FjYRjTSwh97mk_3D).

### Survey participation

Cancer patients aged 18 or over were eligible to participate. A hybrid model of data collection was followed including; Electronic SMS messages, social media platforms (WhatsApp and Facebook), and face-to-face interaction with patients. During their routine clinical visit, the research assistant explained the study's purpose to the patient, and once they consented to participate, they were provided the data collection link. To avoid redundancy and ensure data quality, participants were only allowed to submit one response.

Using the SurveyMonkey sample size calculator the recommended sample size should be 306 participants, based on the following assumption: alpha=0.05, Power 95%, using an estimated population size of 1,500 patients visiting the breast, lung, leukemia, lymphoma, and colon outpatient clinics during the survey study. Nevertheless, over the study period, we were able to collect responses from 441 cancer patients (https://www.surveymonkey.com/mp/sample-size-calculator/).

### Independent variables

The survey questions were designed to learn about cancer patients' attitudes toward SARS-CoV-2 vaccines, discover the primary source of SARS-CoV-2 vaccine information for cancer patients, and investigate current physician practices for SARS-CoV-2 vaccine counseling as well as compare perception and attitude between the pro-vaccine and anti-vaccine groups of patients.

This survey consisted of 29 closed-format questions divided into five sections. (a) Four questions about the demographic features of the participants, such as age, gender, income, and education level. (b) Three questions regarding the patients' medical conditions, such as the type of cancer, treatment status, and current treatment. (c) Seven questions about vaccination history, with a focus on the flu vaccine and the intention to be vaccinated against COVID-19. (d) Nine questions on vaccine evolution, safety, and importance; and (e) Six questions about COVID-19 information. It was necessary to conduct a pilot study to assess the survey's questionnaire validity and internal consistency. Thirty cancer patients were chosen at random and were not included in the study. A panel of specialists was assembled to review the tool's validity (two physicians, two nurses, a psychosocial support technician, a research assistant, and a survey specialist), and minor language changes were made as a result. Furthermore, we calculated internal consistency and a Cronbach alpha test on the pilot sample, and the result was 0.729, indicating good reliability. The survey and consent form was approved by the Research Ethics Committee (IRB) of King Hussein Cancer Center (IRB # 21 KHCC 053). A translated copy of the survey is provided in the supplementary data (Supplementary Data 1).

### Data analysis

To collect data, the questionnaire was uploaded to SurveyMonkey, and to ensure quality, it was set to receive only one response from each device to avoid redundancy. The amount of missing data was negligible, and the research team accepted up to 5% of missing data. Categorical data were summarized in tables as proportions and percentages. Statistical analysis was performed using SPSS 26 (IBM, New York, USA). Descriptive analysis was done for all variables, Pearson Chi-square tests of association, and Fisher Exact measured association among categorical and continuous variables respectively. A *p*-value of < 0.05 was defined as the level of statistical significance. Data were anonymously collected, stored, and analyzed in compliance with the General Data Protection Regulations.

## Results

### Patients' characteristics

A total of 441 patients participated in the study and filled out the questionnaire. Females made up 288 (65.3 %) of the participants, while males made up 153 (34.7 %). Almost half of the participants, 217 (49.2%), were aged between 41 and 60 years, 82 (18.6%) age was between 61 and 70 years, and a minority, 37 (8.4%), were aged between 20 and 30 years. About one-third of participants 150 (33.8 %) have university degrees, followed by 124 (27.4%) who have completed secondary education, while around 47 (10.7 %) of them are below primary education. More than half of the participants had salaries below $705 (251 (57.3%), while 103 (23.6%) had a monthly income between $705.1 and $1,410, and 45 (10.3%) received a salary between $1,410.1 and $2,116. Finally, only 38 (8.7%) have monthly income more than $2,116 [38 (8.7%)] (Table 1).

**Table 1:** The demographic characteristics of the participants.

**Demographic** ** characteristics**	**Response**	***N*** **(%)**
Gender	Male	153 (34.7%)
	Female	288 (65.3%)
Age group	20–30	37 (8.4%)
	31–40	66 (15%)
	41–50	109 (24.7%)
	51–60	108 (24.5%)
	61–70	82 (18.6%)
	> 71	39 (8.8%)
Educational level	Below primary education	47 (10.7%)
	Secondary education	124 (27.9%)
	College	80 (18.1%)
	University	150 (33.8%)
	Post graduate	43 (9.5%)
Monthly income	Less than $423	124 (28.3%)
	$ 423.1–$705	127 (29%)
	$ 705.1–$1,410	103 (23.6%)
	$ 1,410.1–$2,116	45 (10.3%)
	More than $2,116	38 (8.7%)
Diagnosis	Breast cancer	202 (40.3%)
	Leukemia	39 (7.8%)
	Lymphoma	55 (11%)
	Lung cancer	46 (9.2%)
	Colon cancer	38 (7.6%)
	Others	80 (16%)
On cancer treatment	Yes	346 (78.5%)
	No treatment (Survivors clinic)	95 (21.5%)
Type of treatment	Chemotherapy	194 (57.6%)
	Radiotherapy	34 (10.1%)
	Others	109 (32.3%)

The majority of recruited subjects were diagnosed with breast cancer, 202 (40.3%), followed by lymphoma, 55 (11%), lung cancer, 46 (9.2%), leukemia, 39 (7.8%), and colon cancer, 38 (7.6%). Three hundred and forty-six (78.5%) were on active cancer treatment, with only 95 (21.5%) survivors (no current treatment) (Table 1).

### Patients' attitudes toward SARS-CoV-2 vaccines

Our data showed that almost two-thirds of 297 (69.2%) did not get COVID-19 infection, while 132 (30.8%) had already been infected at the study time. Of the 321 cancer patients who registered for the SARS-CoV-2 vaccine, of them; 296 (92.2%) had already received their first dose of the vaccine at the time of the survey, and 287 (97.6%) of participants were planning to take the second dose (Table 2). Moreover, our data demonstrated a significant association between cancer patients who got the flu vaccine and those who took the first dose of SARS-CoV-2 vaccines (*p* **<** 0.05) (Table 3).

**Table 2:** Questions that reflect patients' attitudes toward SARS-CoV-2 vaccines.

**Questions**	**Response**	***N*** **(%)**
Do you have the flu vaccine in 2020?	Yes	108 (25.2%)
	No	321 (74.8%)
Do you think the flu vaccine will protect you from SARS-CoV-2?	Yes	84 (19.6%)
	No	345 (80.4%)
Have you been infected by SARS-CoV-2?	Yes	132 (30.8%)
	No	297 (69.2%)
Have you registered for the SARS-CoV-2 vaccine?	Yes	321 (75.4%)
	No	105 (24.6%)
Did you take the first COVID-19 vaccine dose?	Yes, I got the first dose	296 (92.2%)
	No, I am still waiting for my schedule	25 (7.8%)
Are you going to take the second dose?	Yes	287 (97.6%)
	No, I suffered from side effects, and I do not want to encounter that again	7 (2.4%)
Are you going to register?	Yes	52 (49.5%)
	No (Anti-vaxxers)	53 (50.5%)

**Table 3:** Association between patients who got the first dose of COVID-19 vaccine and who got the flu vaccine and their opinion on the effectiveness of the COVID-19 vaccine.

		**Got the first dose of COVID-19 vaccine**		**Total**	***p-value***
		Yes	Still waiting		<0.05
Vaccinated against flu vaccine in 2020	Yes	97 (97.8%)	2 (2.2%)	99	
	No	199 (89.6%)	23 (10.4%)	222	
Do you think the vaccine will	Yes will decrease symptoms	132 (92.3%)	11 (7.7%)	143	<0.05
	Will decrease infection	125 (94.7%)	7 (5.3%)	132	
	Will not do anything	18 (75.0%)	6 (25.0%)	24	

**Figure 1:**
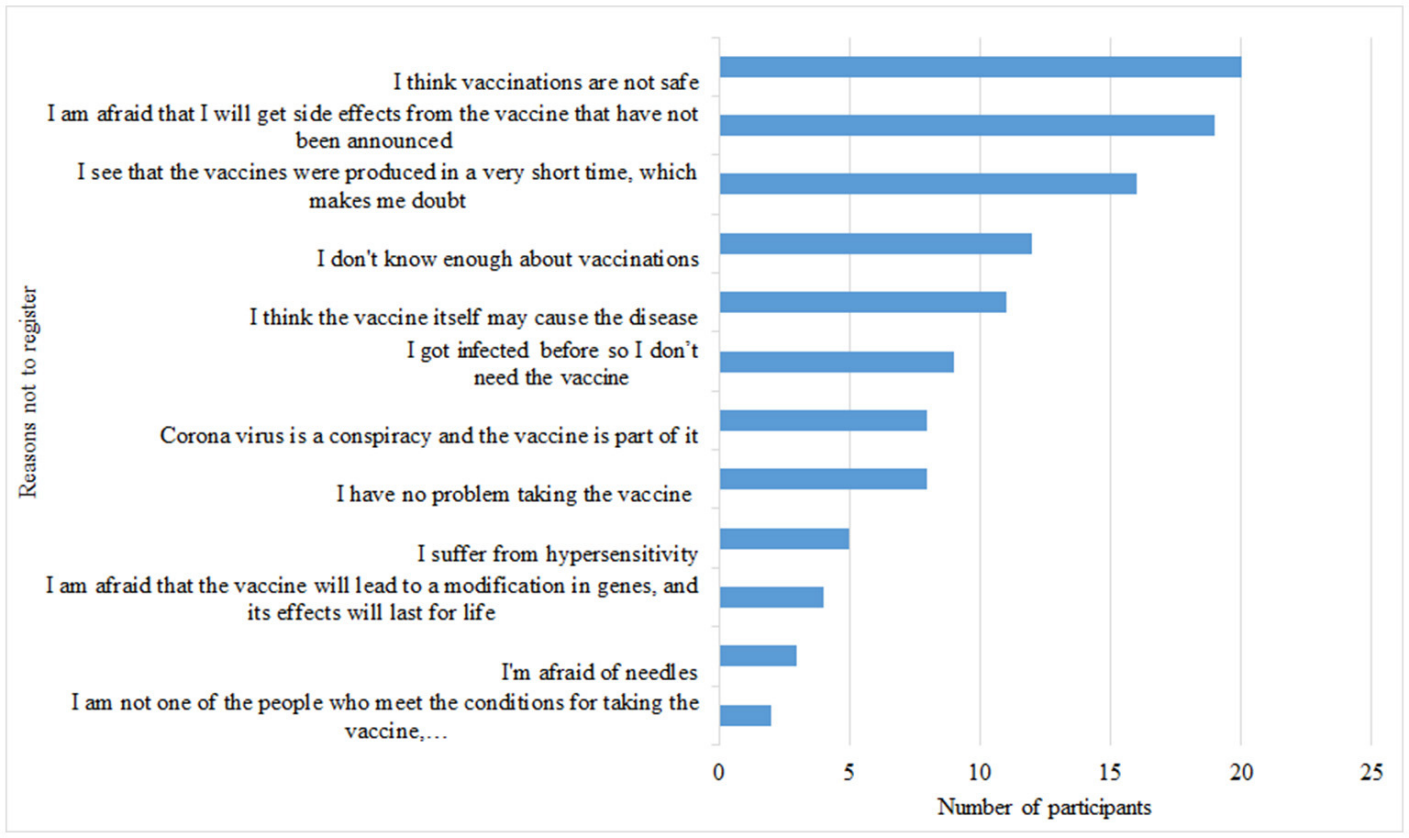
Reasons for not registering for the COVID-19 vaccine. Participants had the option of choosing more than one reason not to register for the vaccine.

Participants who did not register *via* the platform to get COVID 19 vaccines 105 (24.6%) reported different reasons for not taking the COVID-19 vaccine. For example, 20 cancer patients did not feel the vaccines were safe, 19 were concerned about unspecified adverse effects, and 16 believed the vaccine was manufactured in a short period, rendering them suspicious (Figure 1).

**Figure 2:**
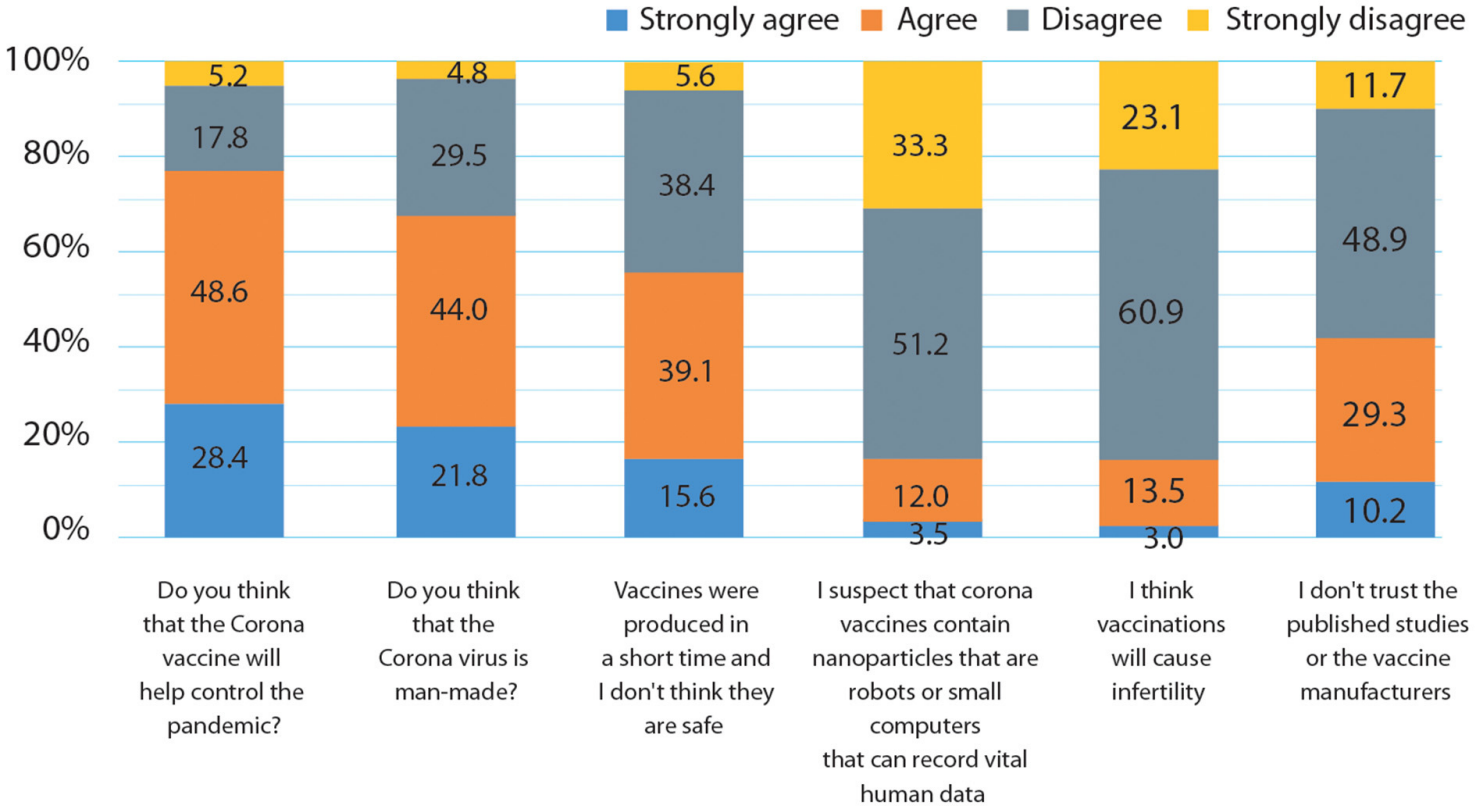
Questions and statements reflect patients' attitudes towards SARS-CoV-2 virus and vaccines.

The COVID-19 vaccination will help control the pandemic, according to 322 (77.6%) of the participants. Two hundred sixty-three (65.8%) participants believed the SARS-CoV-2 virus was created by humans, whereas 137 (34.2%) disagree. Half of the participants, 221 (54.7%), considered that the SARS-CoV-2 vaccinations are unsafe because they were developed in such a short period. The COVID-19 vaccines do not contain nanoparticles that are robots or miniature computers that may record essential human data, according to 338 (84.5%) survey participants. Similarly, 335 (84%) of those surveyed disagree that vaccinations cause infertility. More than half of the participants 244 (60.6%) disagree that published studies and vaccine manufacturers are unreliable (Figure 2).

Most of the participants, 285 (71.8%), believed that the vaccine will protect them for a short time, while 25 (6.3%) only thought that the vaccine will protect them for a lifetime. Furthermore, a vast majority of the participants, 186 (46.9%), thought that the vaccine will reduce the disease symptoms, but will not protect them from being infected, and 159 (40.1%) patients believed it would reduce and protect against COVID-19 infection. Almost half of the participants, 202 (50.9%), thought that they had enough information regarding the vaccine. Nevertheless, 141 (35.5 %) of the cancer patients indicated that they preferred attending awareness lectures on COVID-19 vaccines (Table 4). Results from this survey demonstrated a significant positive association between participants who thought that COVID-19 vaccines would protect them from infection and reduce the signs and symptoms of the disease and those who got the SARS-CoV-2 vaccine (*p* < 0.05) (Table 3).

**Table 4:** Patients' opinions toward COVID-19 vaccines.

**Questions**	**Response**	***N*** **(%)**
Do you think that the vaccine will protect against infection with the Coronavirus?	Yes, it will protect for a short time	285 (71.8%)
	Yes, it will protect for the lifetime	25 (6.3%)
	No, it will not provide protection	87 (21.9%)
Do you think that the corona vaccine will only help relieve the symptoms of infection?	Yes, the vaccine will only help reduce the symptoms of the disease without protecting me from infection	186 (46.9%)
	Yes, the vaccine will help protect me from infection and will also help to reduce the risk of infection	159 (40.1%)
	No, it will not help relieve the symptoms of the disease and will not protect me from infection	52 (13.1%)
Do you think you have enough information about Corona (SARS-CoV-2) vaccines?	Yes	202 (50.9%)
	No	195 (49.1%)
Would you like to attend an awareness lecture on Corona (SARS-CoV-2) vaccines?	Yes	141 (35.5%)
	No	256 (64.5%)

### Patients' knowledge sources and attitudes on the COVID-19 pandemic and SARS-CoV-2 vaccines

**Figure 3:**
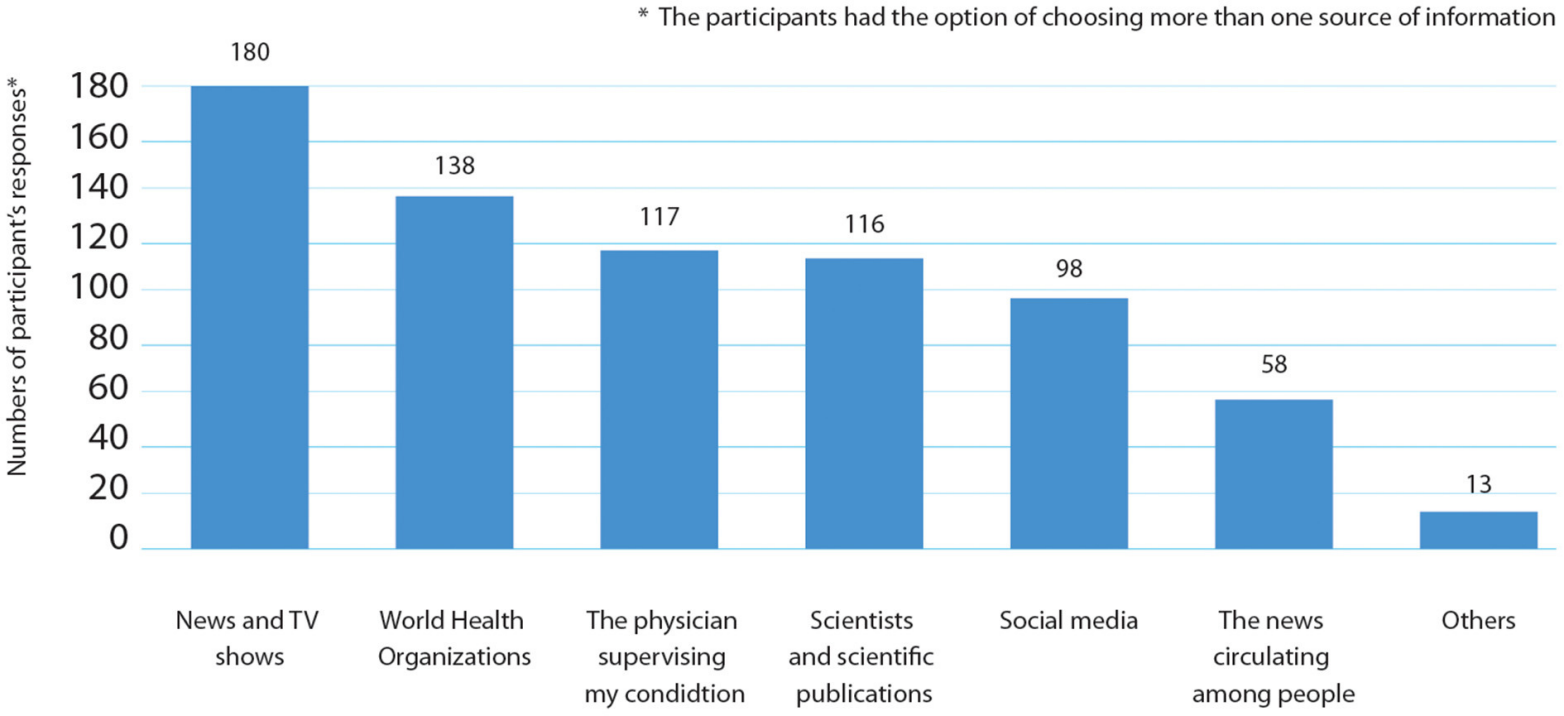
Personal stated sources of information about SARS-CoV-2) vaccines declared by cancer patients.

The sources of information about the SARS-CoV-2 virus vary, as shown in Figure 3; for instance, a substantial number of patients (180/441) got their information from News and TV shows, and (138/441) got their information from the world health organization sites. Moreover, 117 and 116 patients said that their main source of information on COVID-19 was from their oncologists or scientific publications respectively. On the other hand, 98 patients claimed that they relied on social media for their information and 58 admitted they trusted the circulating news to gather the information about the COVID-19 pandemic and vaccines. The participants could choose more than one answer; hence, no percentage was calculated for each subgroup.

There was a positive correlation between patients who got the COVID-19 vaccine and stated that they had enough knowledge about COVID-19 [162/299 (54.2%)] compared to the participants who thought they had good information about the COVID-19 vaccines but did not register for the vaccine [40/98 (40.8%)], *p* < 0.05.

### The impact of current physician practices for SARS-CoV-2 vaccine counseling on the vaccination decision-making of cancer patients

**Figure 4:**
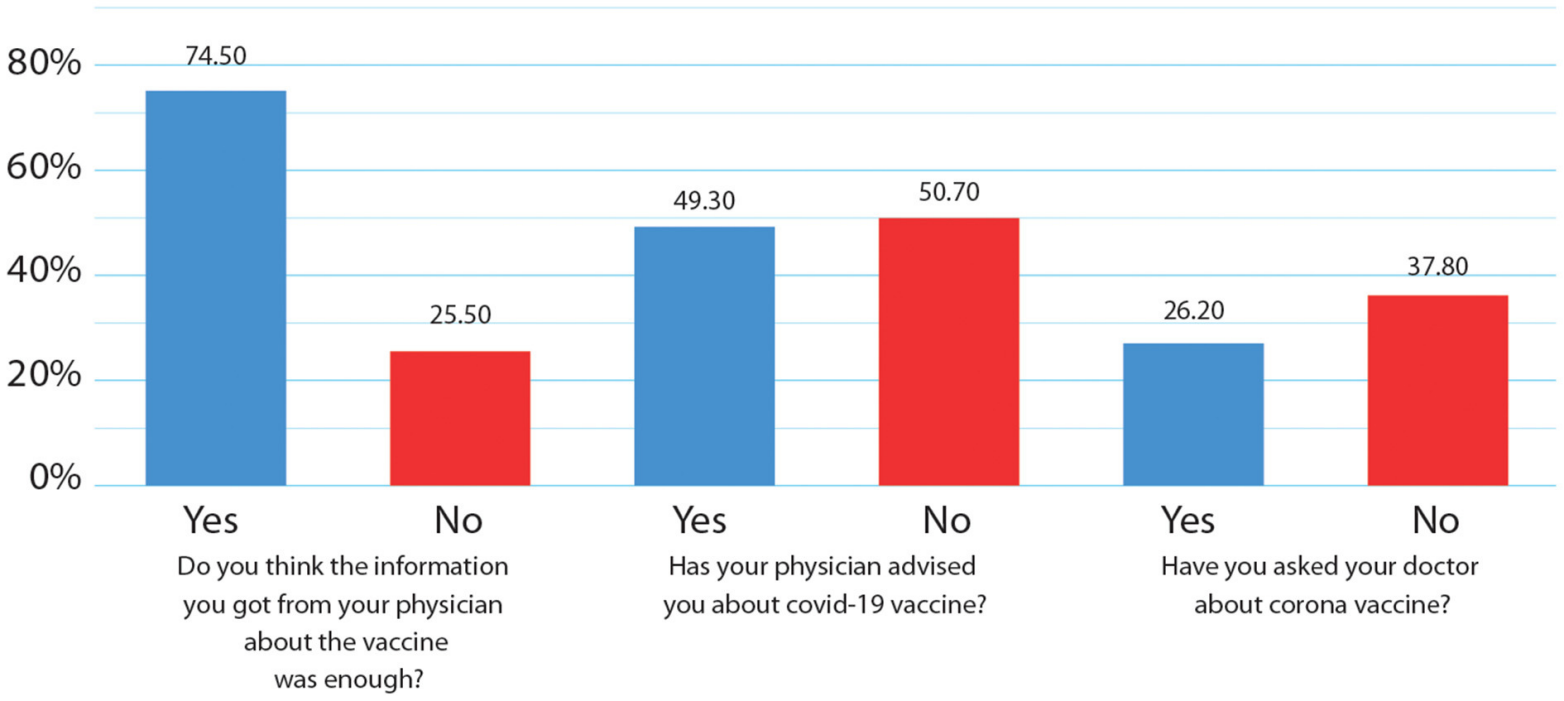
Current physician practices concerning counseling on SARS-CoV-2 vaccines.

Figure 4 shows the physicians' and patients' practices concerning counseling on SARS-CoV-2 vaccines. Almost half of the participants, 201 (49.3%), indicated that their physicians counseled them about the advantages of SARS-CoV-2 vaccines. Although 190 patients (74.5%) thought the vaccine information they received from their oncologists was sufficient, a chi-square test of independence revealed that there was no significant association between claiming to have received sufficient information from their oncologists and their actual registration and willingness to take the COVID-19 vaccine [X^2^ (2, *N* = 255) = 2.2, *p* > 0.05]. Moreover, 152 (37.8%) patients did not ask their physicians about the vaccine, and 141 (35.5%) showed interest to attend awareness lectures about COVID-19 vaccines.

### Patients opposing taking COVID-19 vaccines (COVID-19 anti-vaxxers)

In our study; 53 patients [53/357 (14.8%)] could be classified as COVID-19 anti-vaxxers (anti-vaccine) because they did not register for vaccines and have no plans to do so (Table 2). We found that there was a significant difference in the perception between the pro-vaccine and anti-vaccine (anti-vaxxers) groups on claims listed in Table 5. For example, 32.6 % (16/53) of the anti-vaxxers participants thought that the COVID-19 vaccine would help contain the pandemic, while 85% (260/305) of pro-vaccine participants thought that the vaccine would control the pandemic (*p* < 0.05). Furthermore, 45.5 % (20/53) of anti-vaccine participants believed that vaccination would result in infertility, whereas 89.2 % (227/305) of pro-vaccine participants did not feel that vaccination would result in infertility (*p* < 0.05). Our data also revealed an association between receiving COVID-19 vaccination and trusting manufacturing companies, with a considerable percentage of anti-vaccine participants (77%) expressing mistrust in these companies (*p* < 0.05) (Table 5).

**Table 5:** Perception to ward COVID-19 vaccine, comparison between the pro vaccine and anti-vaccine (anti-vaxxers) groups in the sample.

	**Pro-vaccine 305**		**Anti-vaccine (anti-vaxxers) 53**		***p*** **-value**
	**Agree**	**Disagree**	**Agree**	**Disagree**	
Do you think that the Corona vaccine will help control the pandemic?	260	47	16	33	<0.05
	84.7%	15.3%	32.6%	67.4%	
I think vaccinations will cause infertility	33	272	20	24	<0.05
	10.8%	89.2%	45.5%	54.5%	
I don't trust the published studies or the vaccine manufacturers	101	205	36	11	<0.05
	33%	67%	76.6%	23.4%	
Do you think that the Corona virus is manmade?	195	108	41	6	<0.05
	64.4%	33.6%	87.2%	12.7%	
Vaccines were produced in a short time and I don't think they are safe	151	155	42	6	<0.05
	49.3%	50.7%	87.6%	12.4%	
I suspect that corona vaccines contain nanoparticles that are robots or small computers that can record vital human data.	34	271	15	31	<0.05
	11.1%	88.9%	32.6%	67.4%	

## Discussion

The world currently faces an “infodemic” (19, 27) regarding sources of information on COVID-19 infection and vaccination. Considering that cancer patients infected with SARS-CoV-2 have a higher risk for complications and higher mortality rates, we chose to investigate their attitudes, knowledge, and practices related to the pandemic (28).

Our data showed that 108 patients (25.2%) acknowledged that they took the seasonal influenza vaccine in 2020, similar to the percentage reported in cancer patients from Cyprus (29). A similar number was found in a study of Jordanian university students, with 28.8% having already gotten the flu vaccine (30).

As for SARS-CoV-2 vaccination, most participants in our study, 296 (92.2%), reported that they had already taken the first COVID-19 vaccine shot, which was the only one available at the time of the survey. These results were in line with other published data showing that cancer patients are more willing to take the COVID-19 vaccine than the influenza vaccine (25). On the other hand, a survey performed by Gheorghe et al. on cancer patients in Romania showed that those patients believed that getting the seasonal influenza vaccine would prevent the spread of SARS-CoV-2, and 27.8% declared that they would not get vaccinated against SARS-CoV-2 if a vaccine would become available in Romania (31).

Similar to earlier studies, our findings revealed that the news, TV shows, and the media, in general, were the most common sources of information on the SARS-CoV-2 virus and the COVID-19 pandemic reported by cancer patients (25, 32–34). However, according to a study from Cyprus, social networks were the most prominent source of information for cancer patients (41.2%), while official government websites were the least popular (8.1%) (29). The supervising oncologist was the third most common source of information in our study regarding the SARS-CoV-2 virus similar to data published by Kelkar et al. (35).

The most crucial data in our study found that participants who obtained advice from their doctors and asked their doctors about Coronavirus were 49 and 26%, respectively, which was similar to a study published in Poland (32).

Although, 51% of the participants believed that they had enough information regarding the COVID-19 vaccine, and 75% of those who asked their doctors about the COVID-19 vaccine reported that they got enough information about the vaccine, 35% were still interested in attending awareness lectures, unlike a previous study performed in Jordan on community members where 85% requested more information about COVID-19 vaccines (36).

Our study is the first in Jordan to assess cancer patients' attitudes and knowledge related to the COVID-19 pandemic. It demonstrates the potential influence of sources of information on the tendency to take the vaccines. Although most cancer patients registered for the COVID-19 vaccine, almost 15% of patients still opposed vaccinations. This opposition is most likely due to reliance on misinformation from social media and TV shows based on survey results. We recommend that physicians utilize telehealth technology as an additional resource to their consultation to communicate with their patients, which is akin to online media. Telehealth technology delivers comprehensive, clear, and high-quality digital services that guide and assist patients in better understanding the benefits of COVID-19 vaccines while also saving time during consultations. In addition, involving other hospital services that focus on patients' physical and mental well-being could aid in offering one-on-one guidance to patients during this vital time of uncertainty.

## Data availability statement

The raw data supporting the conclusions of this article will be made available by the authors, without undue reservation.

## Ethics statement

The Institutional Review Board approved this study of King Hussein Cancer Center, Amman, Jordan Center (IRB # 21 KHCC 053) on August 19, 2021. The questionnaire was fully anonymized to protect participants' privacy, and only the collected data were used for analyses and statistical tests. The patients/participants provided their written informed consent to participate in this study.

## Author contributions

Concept and study design and review and editing: LS and MS. Literature search and data collection: LS, SB, and MA. Data analysis, figures, tables, and interpretation of the data: LS and KA. Data validation, visualization, and writing—original draft: LS. All authors approved the final version of the manuscript.

## Funding

Partially funded by the Scientific Research and Innovation Support Fund at the Ministry of Higher Education and Scientific Research (MPH/1/06/2021).

## Conflict of interest

The authors declare that the research was conducted in the absence of any commercial or financial relationships that could be construed as a potential conflict of interest.

## Publisher's note

All claims expressed in this article are solely those of the authors and do not necessarily represent those of their affiliated organizations, or those of the publisher, the editors and the reviewers. Any product that may be evaluated in this article, or claim that may be made by its manufacturer, is not guaranteed or endorsed by the publisher.
