# Supplementary material for: An update on the impact of SARS-CoV-2 pandemic public awareness on cancer patients' COVID-19 vaccine compliance: Outcomes and recommendations
Source: Front Public Health. 2022 Jul 22;10:923815. doi: 10.3389/fpubh.2022.923815 (PMC9354075; doi:10.3389/fpubh.2022.923815)
Supplement: Supplementary file 1 [file Data_Sheet_1.PDF]

Supplement 1B

Cancer Patients Poll on Corona Vaccines

The world is suffering from the Corona pandemic (Covid-19), which continues to cause great global losses due to high rates of infected people, and high mortality rates which increases the level of global burden on healthcare providers, infrastructure of hospitals and health institutions, as well as economic and social losses.

In this questionnaire, we aim to survey about the practices of cancer patients towards the vaccinations that resulted against the Corona virus, and the extent of their desire and acceptance to register to take these vaccines. This survey is expected to take a maximum of 10 minutes from your time, and it is not expected to cause you any negative feelings. Note that your participation is voluntary and you are free to withdraw from the research at any time, knowing that if you do not participate, it will not affect the health services provided to you.

Note: For assurance. This survey does not contain any names or identifier about the participant. Therefore, information confidentiality is guaranteed for the participants in this survey. All data entered will be used in scientific research only.

If you have any questions regarding this study, you may contact the primary investigator Dr. Lina Souan at any time, on the phone number 1414

This study has been reviewed and approved by the Research Ethics Review Committee of the King Hussein Cancer Centre. It is known as the "Institutional Committee" in accordance with the regulations of the Hashemite Kingdom of Jordan. This committee is responsible for protecting your rights and interests as a participant in the research study.

You can contact the office of the Institutional Committee at: 0096265300460 Sub-section 1669 or contact the Chairman of the Institutional Committee on: 1308 or e-mail communication: IRBOffice@KHCC.JO in any of the following cases:

- If you have a query, it's not answered by the study team.
- If you have concerns or complaints about the study or the study team.
- If you have inquiries about your rights as a study participant.
- If you can't call the study team.
- If you want to get additional information or make observations about the study.

Do you accept to participate in this survey?	Yes, I consent to participate.<br>No, I refuse to participate.

Research Office Use Only
Application No.:

Supplement 1B

Cancer Patients Poll on Corona Vaccines

1. Gender	Male
	Female
2. Age Group	20-30
	31-40
	41-50
	51-60
	61-70
	> 71
3. Educational level	Below primary education
	Secondary education
	College
	University
	Post graduate
4. Income	Less than 300 JOD
	300-500 JOD
	500-1000 JOD
	1000-1500 JOD
	More than 500 JOD
5. Diagnosis	Breast Ca
	Leukemia
	Lymphoma
	Lung Ca
	Colon Ca
	Others
6. Are you on active treatment	Yes
	Survivors clinic
7. Type of treatment	Chemotherapy
	Radiotherapy
	Others
8. Have you got flu vaccine in 2020?	Yes
	No
9. Do you think flu vaccine will protect you from COVID-19?	Yes
	No
10. Have you got infected from COVID-19?	Yes
	No
11. Have you registered for COVID-19 vaccine?	Yes
	No
12. If your answer to question 11 was yes: Have you got the first dose?	Yes, I got the first dose
	No, I am still waiting for my schedule

Supplement 1B

Cancer Patients Poll on Corona Vaccines

13. If your answer to question 12 was yes:<br>Are you going to take the second dose?	Yes
	No, I suffered from side effects and I don't want to encounter that again
14. If your answer to question 11 was NO:<br>Are you going to register?	Yes
	No
15. If your answer to question 14 was NO:<br>Reasons not to register	I have no problem taking the vaccine
	I got infected before so I don't need the vaccine
	I am afraid that I will get side effects from the vaccine that have not been announced
	I don't know enough about vaccinations
	I think vaccinations are not safe
	I see that the vaccines were produced in a very short time, which makes me doubt
	I'm afraid of needles
	Corona virus is a conspiracy and the vaccine is part of it
	I am afraid that the vaccine will lead to a modification in genes, and its effects will last for life
	I think the vaccine itself may cause the disease
	I suffer from hypersensitivity
	I am not one of the people who meet the conditions for taking the vaccine, as I am pregnant or breastfeeding
16. Based on your personal opinion kindly answer the following questions.<br>I. Do you think that the Corona vaccine will help control the pandemic?	Strongly Agree<br>Agree<br>Disagree<br>Strongly Disagree
II. Do you think that the Corona virus is man-made?	Strongly Agree<br>Agree<br>Disagree<br>Strongly Disagree
III. Vaccines were produced in a short time and I don't think they are safe	Strongly Agree<br>Agree<br>Disagree<br>Strongly Disagree
IV. I suspect that corona vaccines contain nanoparticles that are robots or small computers that can record vital human data.	Strongly Agree<br>Agree<br>Disagree<br>Strongly Disagree

Supplement 1B

Cancer Patients Poll on Corona Vaccines

V. I think vaccinations will cause infertility	Strongly Agree<br>Agree<br>Disagree<br>Strongly Disagree
VI. I don't trust the published studies or the vaccine manufacturers	Strongly Agree<br>Agree<br>Disagree<br>Strongly Disagree
17. Did your doctor advise you about Corona vaccines?	Yes<br>No
18. Have you asked your doctor about corona vaccines?	Yes<br>No
19. If your answer to question 18 was YES:<br>Do you think the information you got from the doctor about the vaccine was enough?	Yes<br>No
20. What are the sources from which you derive your information about Corona (Covid-19) vaccines?	The doctor supervising my condition
	Scientists and scientific publications
	News and TV Shows
	Social Media
	The news circulating among the people
	world health organizations
	Otherwise, please specify
21. Do you think that the vaccine will protect against infection with the Corona virus?	Yes, it will provide protection for a short time
	Yes, it will provide protection for life
	No, it will not provide protection
22. Do you think that the corona vaccine will only help relieve the symptoms of infection?	Yes, the vaccine will help protect me from infection but it will NOT protect from infection
	Yes, the vaccine will help protect me from infection and will also help to reduce the risk of infection
	No, it will not help relieve the symptoms of the disease and will not protect me from infection
23. Do you think you have enough information about Corona (Covid-19) vaccines?	Yes<br>No
24. If your answer to question 23 was NO:<br>Would you like to attend an awareness lecture on Corona (Covid-19) vaccines?	Yes<br>No

Supplement 1B

Cancer Patients Poll on Corona Vaccines

This study has been reviewed and approved by the Research Ethics Review Committee of the King Hussein Cancer Centre which is also known as the "Institutional Committee" in accordance with the regulations of the Hashemite Kingdom of Jordan. This committee is responsible for protecting your rights and interests as a participant in the research study. You can contact the office of the Institutional Committee at: +962 6 5300460 subdivision 1669 or contact the Chairman of the Institutional Committee on: 1308 or e-mail communication: IRBOffice @ KHCC.JO in any of the following cases:

If you have a query that hasn't been answered by the study team.
If you have concerns or complaints about the study or the study team.
If you have inquiries about your rights as a study participant.
If you can't call the study team.
If you want to get additional information or make observations about the study.
